# Acute pulmonary embolism in a child following SARS-CoV-2 infection: a case report

**DOI:** 10.11604/pamj.2021.38.125.27954

**Published:** 2021-02-04

**Authors:** Amal El Ouarradi, Nabila Chekhlabi, Mahassine Elharras, Ilham Bensahi, Sara Oualim, Fatimazahr Merzouk, Salma Abdeladim, Mohammed Sabry, Nouzha Dini

**Affiliations:** 1 Cardiology Department, Mohammed VI University of Health Sciences, Cheikh Khalifa Hospital, Casablanca, Morocco,; 2 Pediatrics Department, Mohammed VI University of Health Sciences, Cheikh Khalifa Hospital, Casablanca , Morocco,; 3 Pediatrics Department, Military University Hospital Center, Mohammed V University of Medicine of Rabat, Rabat, Morocco

**Keywords:** Pulmonary embolism, thromboembolism, COVID-19 children, SARS-CoV-2 antibody, case report

## Abstract

In children, coronavirus disease 2019 infection is rarely symptomatic. Severe forms with respiratory distress are rare, thromboembolic complications are exceptional. We report a rare case of a 14 years old girl with severe acute respiratory syndrome coronavirus 2 (SARS-CoV-2) infection who was admitted to the hospital for bilateral pulmonary embolism with intracardiac thrombus. The girl progressed well on anticoagulation.

## Introduction

The coronavirus pandemic, which affects all age groups, appears to be less severe in children. Patients under 18 years of age represent only 2% of severely affected patients. Thromboembolic complications remain exceptional in this age group [1]. We present a rare case of an adolescent with massive pulmonary embolism following a coronavirus disease 2019 (COVID-19).

## Patient and observation

**Figure 1:**
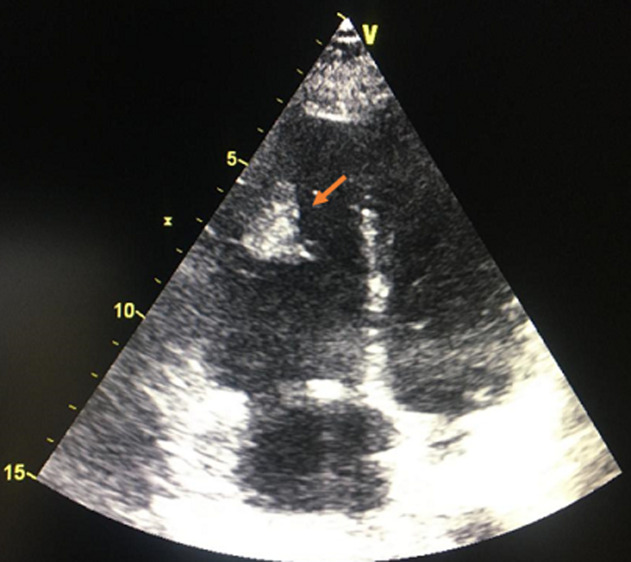
echocardiography - an apical four chamber view showing the presence of a mass on the lateral wall of the right ventricle (arrow)

**Figure 2:**
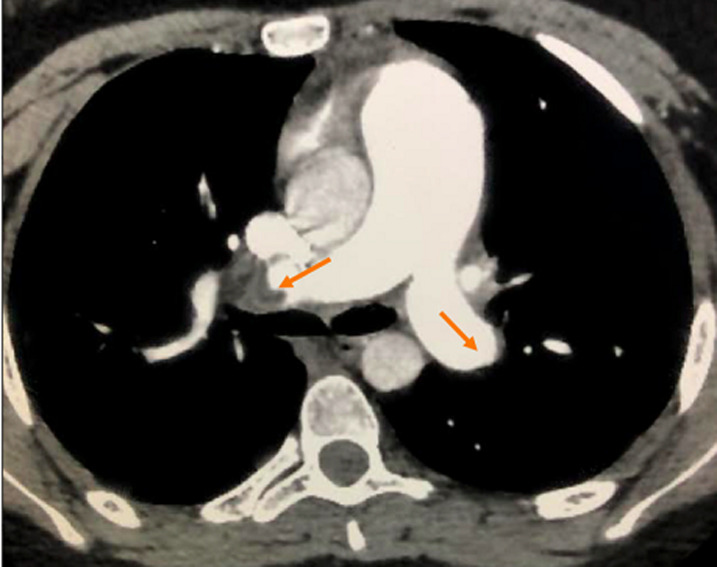
computed tomography angiogram demonstrates bilateral filling defects in the main pulmonary arteries (red arrows)

**Figure 3:**
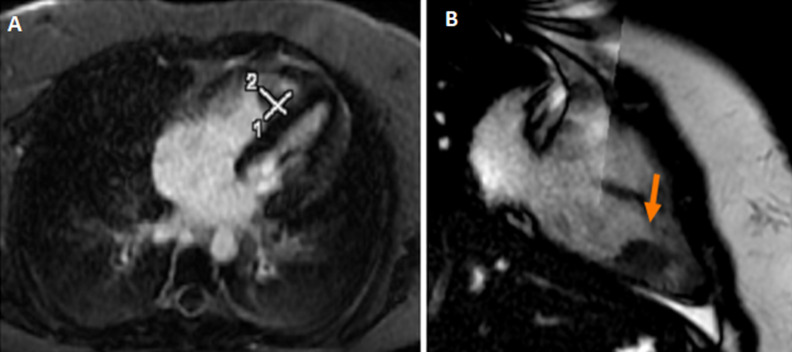
cardiac magnetic resonance imaging; thrombus in the lateral wall of the right ventricle (red arrow)

A 14-year-old teenager with no particular pathological history, apart from moderate obesity with a body mass index (BMI) of 31 kg/m^2^, was admitted for a class IV dyspnea according to the New York Heart Association (NYHA) classification with sudden chest pain. The symptomatology started 3 weeks before admission, with an influenza syndrome and a dry cough complicated by an effort dyspnea. It should be noted that her parents were undergoing COVID-19 treatment. The patient consulted a general practitioner, who prescribed treatment with antibiotics, oral corticosteroids and inhaled bronchodilators. The evolution was characterized by a progressive worsening of dyspnea and the appearance of palpitations and chest pain. The clinical examination showed a pale, anxious, apyretic patient, with tachycardia at 125 beats per minute (BPM), a blood pressure at 110/70 mmHg and an arterial saturation in ambient air at 90%. Auscultation found regular tachycardia with no audible murmur, no signs of right or left heart failure, no sign of deep vein thrombosis. The initial biological assessment noted a normal blood count and hemostasis, D-dimer level was very high at 3520 ngFEU/ml and an inflammatory syndrome with a sedimentation rate at 28 mm and C-reactive protein at 26 mg/l, with negative procalcitonin was found (Table 1). Echocardiography found signs of an acute pulmonary heart with a very dilated right ventricle. It showed a mass on its side wall (Figure 1), a pulmonary hypertension at 50 mmHg, with a paradoxical septum. The systolic function of the right and left ventricle was preserved. The computed tomography (CT) angiogram of the chest showed a bilateral massive pulmonary embolism (Figure 2) without parenchymal lesions. The cardiac magnetic resonance imaging (MRI) confirmed the thrombotic nature of the mass and eliminated its tissue character. The systolic function of the right ventricle was preserved (Figure 3). The patient was hospitalized in an intensive care unit. Anticoagulation with unfractionated heparin was started as continuous infusion to target an APTT of 2 to 3 times control. Thrombolysis was not administered as her hemodynamic status was stable. As part of the etiological assessment of her pulmonary embolism, a venous Doppler of the lower limbs with an abdominal ultrasound was normal. Biological assessment for thrombophilia (C protein, S protein, antithrombin) or autoimmune disease (anti-cardiolipin antibody immunoglobulin M (IgM) and immunoglobulin G (IgG), anti-nuclear antibody, anti-DNA antibody) was negative as well. SARS-CoV-2 nasopharyngeal polymerase chain reaction (PCR) was negative but SARS-CoV-2 antibody was positive (IgG). Clinical improvement with regression of acute pulmonary heart disease, pulmonary hypertension and right ventricular mass was noted. The patient was discharged on Antivitamin K and aspirin at anticoagulant dose. After two months, there was a clear clinical improvement with disappearance of the mass in the right ventricle.

**Table 1:** serum laboratory values from the initial presentation

Laboratory	Value patient	Normal value
White blood cell count	11.25 10^3^/mm^3^	4.5-13
Neutrophil polynuclear	6.91 10^3^/mm^3^	1.5-7.2
Hemoglobin	13.1 g/dl	11.3-16
Platelet	274 10^3^/mm^3^	160-430
Sedimentation rate	28 mm	< 13
C-reactive protein	26 mg/l	0.1-2.8
Fibrinogen	2.8 g/dl	2-4.5
NT-pro BNP	1531 pg/ml	< 300
Ultrasensitive T troponins	0.024ng/ml	< 0.14
Ferritin	70 ng/ml	15-80
Procalcitonin	0.065 ng/ml	< 0.5
D-dimer	3520 ngFEU/ml	< 500
Creatinin	5.5 mg/l	4.6-7.7

## Discussion

The 2019 coronavirus disease has been considered, since March 2020, as a real global pandemic. Clinical features of COVID-19 include fever, cough, fatigue, diffuse myalgia, anosmia, and sometimes digestive disturbances. Dyspnea is observed in about a fifth of patients [2]. Severe cases are characterized by acute respiratory distress syndrome, metabolic acidosis, systemic inflammation, coagulation dysfunction, and organ failure [3]. In addition to the immobility induced by asthenia and dyspnea, COVID-19 predisposes to systemic inflammation which increases the risk of deep vein thrombosis [4] and pulmonary embolism. Pulmonary embolism was observed in 16.7 to 47% of patients admitted to the intensive care unit despite the systematic use of thromboprophylaxis [5]. According to various publications, COVID-19 appears to infect children less frequently and cause milder symptoms with much lower death rates compared to adults [6]. A pediatric incidence, between 0.8% and 2%, has been recorded in several series around the world [7,8]. Recent international publications on SARS-CoV-2 recruit more adults than children. Thromboembolic complications associated with COVID-19 are exceptional in children [9]. The few cases reported in recent literature have risk factors that have probably contributed, in addition to the COVID-19 infection, to its thrombotic complications, particularly obesity, pregnancy and recent surgery [10,11]. The mechanisms by which thromboembolic complications arise in SARS-CoV-2 infection may be related to several factors [9,12-15]. The cytokine storm caused by the COVID-19 viral infection induces the expression of tissue factors on the surface of endothelial cells and leukocytes. These tissue factors are the primary initiator of the blood coagulation cascade and are a major contributor to the hypercoagulated state of COVID-19 infection. Inflammatory cytokines can also trigger the release of von Willebrand factor from the endothelium, causing thrombotic microangiopathy. Finally, the concentrations of heparin-like vascular molecules are reduced by inflammation, interfering with the natural pathways of anticoagulants. The second potential mechanism is virus-induced endothelial dysfunction. SARS-CoV-2 is able to directly infect vascular endothelium by entering cells via receptors for the enzyme angiotensin, resulting in the massive release of plasminogen activators and inhibition of fibrinolysis. The third hypothetical mechanism is complement activation in viral pneumonia. Other clinical factors, such as hypoxemia, hyperthermia, and hypovolemia, may also increase the state of hypercoagulation in patients treated with COVID-19. The difference in frequency of thromboembolic events between children and adults may be related to the difference in receptors in the renin-angiotensin system between the two age groups and the modification of inflammatory responses to pathogens [12]. Pulmonary embolism in adults appears to be associated with more extensive lung damage, it is more common in patients on invasive ventilation with comorbidity. Thoracic angiography is the first examination to order, it confirms the diagnosis of pulmonary embolism, specifies its location and looks for signs of seriousness. Embolism is often bilateral and segmental [15]. Treatment for pulmonary embolism or thromboembolic events in children and adolescents is similar to that in adults. Therapeutic anticoagulation is the cornerstone of the management of pulmonary embolism. Treatment modalities vary depending on the severity of the embolic disease: medical management alone with oral anticoagulants or heparin is indicated in patients with stable hemodynamics. In children, only the antivitamins K can be used. The newer oral anticoagulants (apixaban, dabigatran, rivaroxaban) are not authorized in this population. For patients with secondary hemodynamic instability or deterioration, systemic thrombolysis is indicated and procedural interventions such as catheter-directed thrombolysis and surgical thrombectomy are possible therapies [15]. The high incidence of thrombotic complications in patients with COVID-19 has led to the introduction of systematic pharmacological prophylaxis in hospitalized adult patients even without other risk factors. In the pediatric population, no prophylaxis has been suggested for COVID-19 infection [16,17]. Aspirin is used in anti-aggregation doses in patients at different stages of infection. It has been associated with a reduction in thrombo-inflammation and a lower rate of clinical complications and hospital mortality [18]. Finally, the search for an underlying etiology associated with the COVID-19 infection is necessary to avoid recurrence and adapt the treatment: search for thrombophilia, neoplasia disease or underlying autoimmune disease such as lupus, Crohn's disease, Behçet's disease or anti-phospholipid syndrome. Emerging reports show that SARS-CoV-2 infection precedes the appearance of various autoimmune and autoinflammatory diseases, including pediatric inflammatory multisystemic syndrome or multisystem inflammatory syndrome in children [19].

## Conclusion

This clinical case highlights the potential for thromboembolic complications during COVID-19 infection in the pediatric population, hence the need for increased vigilance with this complication. Etiological assessment should be performed to detect underlying autoimmune and auto-inflammatory diseases is essential. An effective prophylaxis dose and close monitoring are discussed in children with COVID-19 and high body mass index.
